# CT Scan-Derived Muscle, But Not Fat, Area Independently Predicts Mortality in COVID-19

**DOI:** 10.1016/j.chest.2023.02.048

**Published:** 2023-03-07

**Authors:** Sophie I.J. van Bakel, Hester A. Gietema, Patricia M. Stassen, Harry R. Gosker, Debbie Gach, Joop P. van den Bergh, Frits H.M. van Osch, Annemie M. W.J. Schols, Rosanne J. H.C.G. Beijers

**Affiliations:** a Department of Respiratory Medicine, NUTRIM School of Nutrition and Translational Research in Metabolism, Maastricht University Medical Center+, Maastricht, the Netherlands; b Department of Internal Medicine, NUTRIM School of Nutrition and Translational Research in Metabolism, Maastricht University Medical Center+, Maastricht, the Netherlands; c Section Acute Medicine, Division of General Internal Medicine, Department of Internal Medicine, CARIM School for Cardiovascular Diseases, Maastricht University Medical Center+, Maastricht, the Netherlands; d Department of Radiology and Nuclear Medicine, Maastricht University Medical Center+, Maastricht, the Netherlands; e Grow School for Oncology and Reproduction, Maastricht University Medical Center+, Maastricht, the Netherlands; f Department of Clinical Epidemiology, VieCuri Medical Centre, Venlo, the Netherlands; g Department of Internal Medicine, VieCuri Medical Centre, Venlo, the Netherlands

**Keywords:** body composition, COVID-19, CT scan, mortality, skeletal muscle

## Abstract

**Background:**

COVID-19 has demonstrated a highly variable disease course, from asymptomatic to severe illness and eventually death. Clinical parameters, as included in the 4C Mortality Score, can predict mortality accurately in COVID-19. Additionally, CT scan-derived low muscle and high adipose tissue cross-sectional areas (CSAs) have been associated with adverse outcomes in COVID-19.

**Research Question:**

Are CT scan-derived muscle and adipose tissue CSAs associated with 30-day in-hospital mortality in COVID-19, independent of 4C Mortality Score?

**Study Design and Methods:**

This was a retrospective cohort analysis of patients with COVID-19 seeking treatment at the ED of two participating hospitals during the first wave of the pandemic. Skeletal muscle and adipose tissue CSAs were collected from routine chest CT-scans at admission. Pectoralis muscle CSA was demarcated manually at the fourth thoracic vertebra, and skeletal muscle and adipose tissue CSA was demarcated at the first lumbar vertebra level. Outcome measures and 4C Mortality Score items were retrieved from medical records.

**Results:**

Data from 578 patients were analyzed (64.6% men; mean age, 67.7 ± 13.5 years; 18.2% 30-day in-hospital mortality). Patients who died within 30 days demonstrated lower pectoralis CSA (median, 32.6 [interquartile range (IQR), 24.3-38.8] vs 35.4 [IQR, 27.2-44.2]; *P* = .002) than survivors, whereas visceral adipose tissue CSA was higher (median, 151.1 [IQR, 93.6-219.7] vs 112.9 [IQR, 63.7-174.1]; *P* = .013). In multivariate analyses, low pectoralis muscle CSA remained associated with 30-day in-hospital mortality when adjusted for 4C Mortality Score (hazard ratio, 0.98; 95% CI, 0.96-1.00; *P* = .038).

**Interpretation:**

CT scan-derived low pectoralis muscle CSA is associated significantly with higher 30-day in-hospital mortality in patients with COVID-19 independently of the 4C Mortality Score.

## Graphical Abstract

**Figure:**
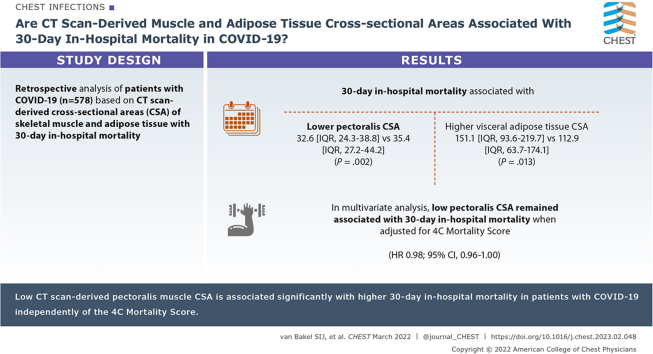

FOR EDITORIAL COMMENT, SEE PAGE 269

**Take-home Points**

**Study Question:** Are CT scan-derived muscle and adipose tissue cross-sectional areas (CSAs) associated with 30-day in-hospital mortality in patients with COVID-19, independent of 4C Mortality Score?

**Results:** In multivariate analyses, low pectoralis muscle CSA was associated with 30-day in-hospital mortality when adjusted for 4C Mortality Score.

**Interpretation:** Low CT scan-derived pectoralis muscle CSA is associated significantly with higher 30-day in-hospital mortality in patients with COVID-19 independently of the 4C Mortality Score.

COVID-19 caused by the SARS-CoV-2 presents with a highly variable disease course varying from asymptomatic disease to severe illness requiring hospitalization, ICU admission, mechanical ventilation, and eventually death.^1^^,^^2^ However, the high prevalence of SARS-CoV-2 infections resulted in very high absolute numbers of severely ill patients requiring hospitalization and high mortality rates of up to 20% to 25% in several European regions, putting a high burden on hospitals and health-care systems worldwide.^3^^,^^4^ Early diagnosis of COVID-19 and identification of patients at high risk for severe illness and mortality are essential for adequate clinical decision-making and managing the large numbers of severely ill patients. For this purpose, chest CT scan imaging was found useful from a very early stage in the pandemic onward.^5^, ^6^, ^7^, ^8^ Based on systematic classification of intrapulmonary abnormalities, these chest CT scans can provide a likelihood of COVID-19 with high diagnostic accuracy.^7^, ^8^, ^9^, ^10^

Next to the pulmonary abnormalities, CT scans contain relevant information on muscle and adipose tissue mass and distribution.^11^ Quantification of muscle cross-sectional area (CSA) at the level of the third lumbar vertebra is considered the reference for estimating whole body muscle mass.^11^^,^^12^ However, analyses at higher vertebral levels available on chest CT scan images, for example, at the level of the first lumbar vertebra or the pectoralis muscle, also recently were validated for assessment of clinically relevant muscle mass.^12^^,^^13^ Additionally, the levels of first and third lumbar vertebrae appeared to be comparable for assessment of adipose tissue mass and distribution.^12^ Therefore, chest CT scans obtained for the diagnosis and assessment of severity of pulmonary involvement also can be used to gain insight into body composition of these patients.

Multiple authors have investigated the possible prognostic value of CT scan-derived body composition parameters on COVID-19 outcomes.^14^, ^15^, ^16^ Methodology and exact anatomic levels at which these parameters were quantified varied among studies. Still, meta-analyses showed that low skeletal muscle mass predicts short-term mortality and high visceral adipose tissue (VAT), but not subcutaneous adipose tissue (SAT), is associated with more severe disease in patients with COVID-19.^14^^,^^15^

Currently, clinical decision-making in the management of patients with COVID-19 is based on vital parameters and blood analyses that are available rapidly and commonly in the ED. With these parameters, a multitude of models predicting adverse outcomes in COVID-19 have been developed. Based on a living systematic review, the 4C Mortality Score was developed and validated by Knight et al^17^ in derivation and validation cohorts of 35,463 and 22,361 patients, respectively, and has been identified as the most extensively validated and best model to predict in-hospital mortality in COVID-19 after 30 days.^17^, ^18^, ^19^ The 4C Mortality Score is a risk stratification score based on the following highly predictive clinical items; sex, age, number of comorbidities, vital signs, blood urea level, and C-reactive protein (CRP) level. Essentially, it combines information on the acute clinical state of the patient (eg, vital signs, blood urea level, and CRP level) and the preexisting condition of the patient (eg, age and number of comorbidities).

Because the CT scan-derived body composition parameters inherently are associated with clinical parameters commonly used to reflect patients’ preexisting conditions, it can be questioned whether CT scan-derived body composition is associated with mortality independent of the 4C Mortality Score. Therefore, this study aimed to evaluate the association of CT scan-derived body composition parameters independent of a validated set of predictive clinical parameters on 30-day in-hospital mortality in patients with COVID-19.

## Study Design and Methods

### Study Design and Population

This was a retrospective, multicenter cohort analysis of patients with COVID-19 from the Maastricht University Medical Centre+ (MUMC+) and the VieCuri Medical Centre in the province of Limburg, The Netherlands. Both cohorts consisted of consecutive adult patients who sought treatment at the ED of the concerning hospital with a primary clinical suspicion of COVID-19 during the first wave of the pandemic and underwent chest CT scan imaging at presentation. All cases of COVID-19 either were confirmed by reverse-transcription polymerase chain reaction testing or had a high clinical likelihood in combination with a COVID-19 Reporting and Data System (CO-RADS) score of ≥ 4 (eg, a high likelihood based on CT scan abnormalities) and no alternative diagnosis.^7^ The MUMC+ cohort consisted of both hospitalized patients and patients who presented at the ED, but were not admitted, whereas the VieCuri cohort consisted of only hospitalized patients. Mortality within 30 days was checked systematically for all patients, regardless of hospitalization status. Because of the retrospective nature of the study, the medical ethics committee of MUMC+ waived ethical approval for this study (Identifier: METC 2020-2230), and therefore, no informed consent was required. Additionally, on discharge from the hospital, patients were informed about the possible use of their (anonymized) data for research purposes. In case patients objected to this, they were excluded from the database.

### CT Scan Image Analysis

**Figure 1:**
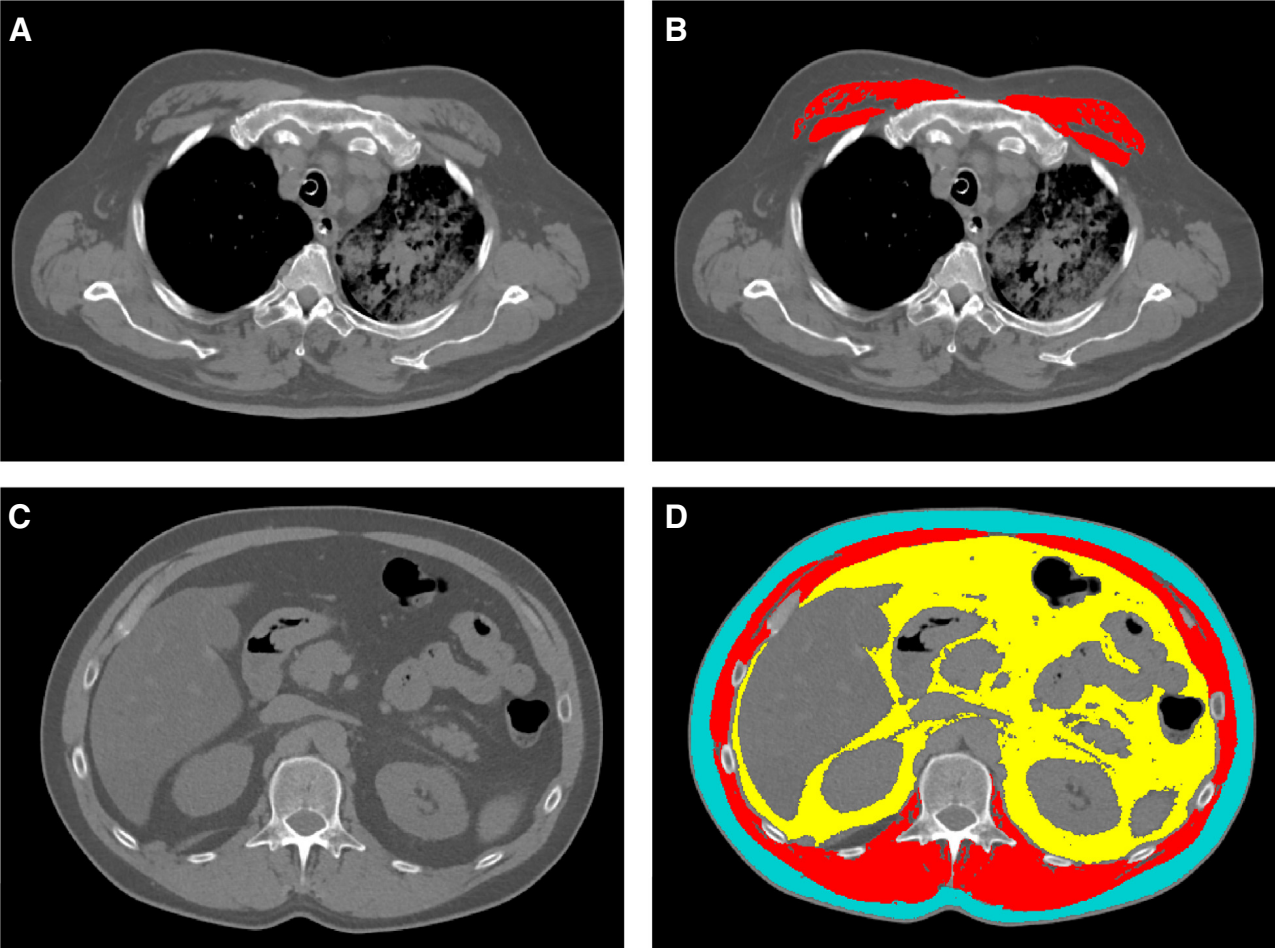
A-D, Representative examples of selected CT scan slices at the fourth thoracic vertebra level (A) with demarcated pectoralis major and minor muscle (B) and at first lumbar vertebra (L1) level (C) with demarcated L1 muscle, visceral, and subcutaneous adipose tissue (D).

Skeletal muscle and adipose tissue parameters were retrieved from routinely obtained chest CT scans at ED presentation or admission. All CT scans were obtained without the application of IV contrast. Total cross-sectional area (CSA) of the pectoralis major and minor muscles was measured bilaterally at the level of the fourth thoracic vertebra. Additionally, CSA of skeletal muscle, VAT, and SAT was demarcated at the level of the first lumbar vertebra (L1). The muscles analyzed at the L1 level included the psoas, erector, spinae, quadratus lumborum, transversus abdominis, external and internal oblique, and rectus abdominis. At both levels, following previously described methods, a single transverse image at the most cranial slide with both vertebral transverse processes clearly visible was used (Fig 1).^12^^,^^13^ If the selected image was of poor quality, had artefacts, or did not fully depict tissue of interest, the specific slice or missing tissue was considered as a missing value and was not analyzed. CSA of these structures were quantified by one trained assessor, blinded to clinical outcomes, based on pre-established Hounsfield units (HU) thresholds (skeletal muscle, –29 to 150 HU; SAT, –190 to –30 HU; and VAT, –150 to –50 HU).^20^^,^^21^ Boundaries were corrected manually when necessary. All analyses were performed with Slice-O-Matic software version 5.0 (Tomovision).

### Clinical Parameters and Outcome Measures

Patient demographics (age, sex, body height, and weight), clinical observations (number of comorbidities, respiratory rate, peripheral oxygen saturation on room air, and Glasgow coma scale score), blood parameters (urea and CRP levels), and information on disease course (ICU or medium care unit admission, mechanical ventilation) were collected retrospectively from the electronic medical records in both institutions. The eight parameters of the 4C Mortality Score were categorized and scored, resulting in a total 4C Mortality Score ranging from 0 to 21.^17^ Additionally, date of presentation at the ED, date of CT scan imaging, date of discharge, and (if applicable) date of death also were retrieved from the medical records.

### Statistical Analyses

Baseline clinical variables of individuals who died in hospital within 30 days and of survivors were compared using the χ^2^ test for categorical variables and the nonparametric Mann-Whitney *U* test for the continuous CT scan-derived variables with skewed distributions. Data are presented as numbers and percentages for categorical variables and medians and interquartile ranges (IQRs) for continuous variables. Missing values of individual components of the 4C Mortality Score were replaced using multiple imputation if values were missing in < 20% of patients. In case more than two components of the 4C Mortality Score were missing, no imputation was performed. Receiver operating characteristic curve analysis was performed to check the area under the receiver operating characteristic curve (AUC) of the 4C Mortality Score. Univariate Cox proportional hazard regression models were applied to assess the association of independent CT scan-derived skeletal muscle mass and adipose tissue CSA with 30-day in-hospital mortality. All parameters with a *P* value of ≤ .1 were considered for inclusion in a multivariate model, adjusted for the 4C Mortality Score, with a forward selection likelihood ratio approach. Multivariate Cox regression analyses then were applied to assess the association of the CT scan-derived parameters adjusted for the 4C mortality score with 30-day in-hospital mortality. Results of the Cox regressions are presented as hazard ratios (HRs) with 95% CIs.

Because the 4C Mortality Score was validated specifically for hospitalized patients, sensitivity analyses were performed investigating the association between CT scan-derived parameters and 30-day in-hospital mortality in hospitalized patients as well as the association with 30-day overall mortality in all patients. Subsequently, potential interactions between CT scan-derived parameters and individual components of the 4C Mortality Score were evaluated using nonparametric Kruskal-Wallis tests. This allowed the construction of categorical variables using age- and sex-specific cutoffs based on the cohort’s IQRs, which were added to an adjusted 4C Mortality Score. Finally, a comparative receiver operating characteristic curve analysis was performed to quantify the added value of CT scan-derived parameters to the 4C Mortality Score. All statistical analyses were performed using SPSS statistical software (SPSS Statistics for Windows version 27.0; IBM). A *P* value of ≤ .05 was considered statistically significant.

## Results

### General Characteristics

**Figure 2:**
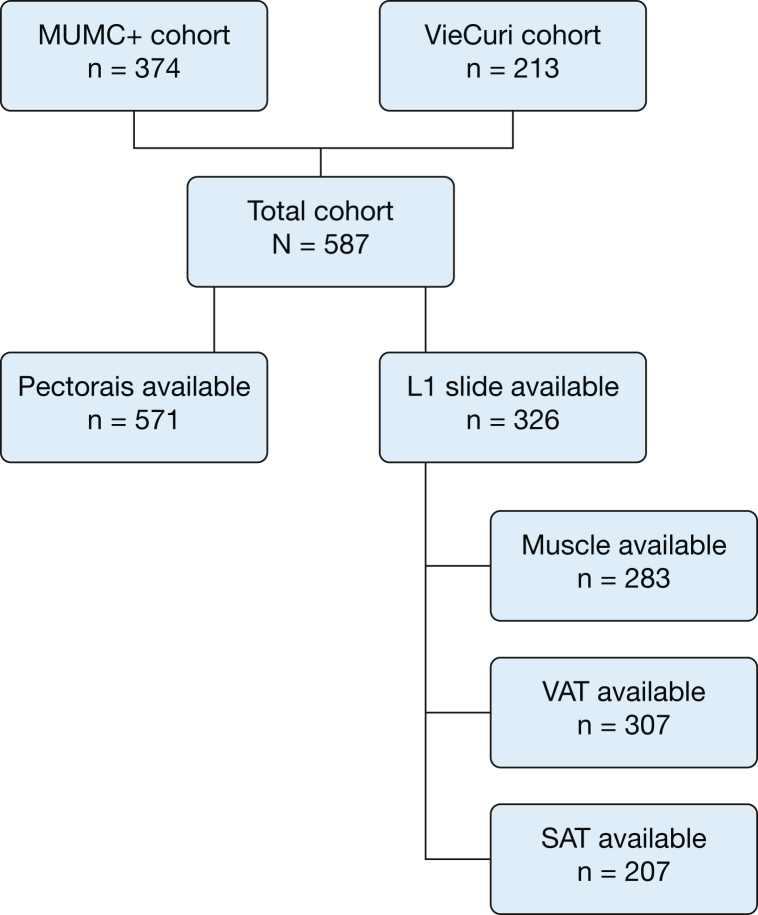
Flow chart showing available CT scan images and tissues of interest. If a selected image was of poor quality, had artefacts, or tissue of interest was not depicted fully, the specific slice or missing tissue was considered as a missing value. L1 = first lumbar vertebra; MUMC+ = Maastricht University Medical Centre+; SAT = subcutaneous adipose tissue; VAT = visceral adipose tissue.

Data from 587 patients were analyzed, including 374 patients from the MUMC+ cohort and 213 patients from the VieCuri cohort (Fig 2), with an admission rate of 82.5% (484 patients). Missing values for urea level (2.7%) and CRP level (0.3%) were imputed. Most patients were elderly men, with most being overweight or obese (Table 1). Within 30 days, 107 patients (18.2%) died in hospital with a median time to death of 6 days (IQR, 3-11 days). An additional 13 patients (2.2%) died outside of the hospital within 30 days. Deceased patients had significantly more comorbidities and scored significantly worse on all clinical and blood parameters of the 4C Mortality Score compared with survivors. In univariate Cox regression analysis, the 4C Mortality Score significantly predicted 30-day in-hospital mortality (HR, 4.6; 95% CI, 3.4-6.3; *P* < .001). The AUC for the 4C Mortality Score was 0.806 (95% CI, 0.765-0.848; *P* < .001).

**Table 1:** General Characteristics

Variable	In-Hospital Survival (> 30 *d*; n = 480 [81.8%])	In-Hospital Death (≤ 30 *d*; n = 107 [18.2%])	*P* Value
Age, y			**< .001**
< 50	49 (10.2)	0 (0)	
50-60	97 (20.2)	5 (4.7)	
60-70	137 (28.5)	7 (6.5)	
70-80	131 (27.3)	48 (44.9)	
> 80	66 (13.8)	47 (43.9)	
Male sex	302 (62.9)	80 (74.8)	**.020**
BMI, kg/m^2^			**.036**
< 20	4 (1.0)	5 (5.2)	
20-25	112 (28.4)	26 (26.8)	
26-30	157 (39.8)	42 (43.3)	
> 30	121 (30.7)	24 (24.7)	
No. of comorbidities			**< .001**
0	139 (29.0)	9 (8.4)	
1	105 (21.9)	17 (15.9)	
≥ 2	236 (49.2)	81 (75.7)	
SpO_2_ < 92%	145 (30.2)	53 (49.5)	**< .001**
Respiratory rate, breaths/min			**.001**
< 20	216 (45.0)	28 (26.2)	
20-30	202 (42.1)	56 (52.3)	
> 30	62 (12.9)	23 (21.5)	
Glasgow coma scale < 15	42 (8.8)	22 (20.6)	**< .001**
Blood urea, mmol/L			**< .001**
< 7	306 (63.7)	34 (31.8)	
7-14	132 (27.5)	49 (45.8)	
> 14	42 (8.8)	24 (22.4)	
Blood CRP, mg/L			**.034**
< 50	170 (36.5)	29 (27.1)	
50-100	137 (28.8)	25 (23.4)	
> 100	173 (34.8)	53 (49.5)	
ICU admission	74 (15.7)	31 (29.0)	**.001**
MC admission	35 (7.1)	10 (9.3)	.470
Mechanical ventilation	80 (18.8)	39 (38.6)	**< .001**

Data are presented as No. (%), unless otherwise indicated. Boldface indicates a *P* value with statistical significance. CRP = C-reactive protein; MC; medium care, min; minute, SpO_2_ = peripheral oxygen saturation on room air.

### CT Image Analyses

Within the total cohort of 587 patients, pectoralis muscle CSA could be determined on 571 scans (97.3%), whereas at the L1 level, VAT CSA could be analyzed on 307 scans (52.3%), muscle CSA on 283 scans (48.2%), and SAT CSA on 201 scans (34.2%) (Fig 2). Patients who died within 30 days showed a significantly lower pectoralis muscle CSA (*P* = .002) and tended to have a lower L1 muscle CSA (*P* = .087) compared with survivors. Additionally, their VAT, but not SAT CSA was significantly higher (*P* = .013 and *P* = .983, respectively) (Table 2). Pectoralis muscle CSA was significantly higher in men (median, 38.8 cm^2^; IQR, 32.0-46.2) compared with that in women (median, 26.5 cm^2^; IQR, 22.1-31.7 cm^2^; *P* < .001) and decreased with increasing age (*P* < .001 for trend).

**Table 2:** CT Scan-Derived Body Composition Parameters

Variable	In-Hospital Survival (> 30 *d*)	In-Hospital Death (≤ 30 *d*)	*P* Value
Pectoralis muscle, cm^2^			
No.	467	104	
Median (IQR)	35.4 (27.2-44.2)	32.6 (24.3-38.8)	**.002**
L1 muscle, cm^2^			
No.	232	51	
Median (IQR)	88.1 (72.1-108.6)	85.7 (66.4-103.2)	.087
L1 VAT, cm^2^			
No.	251	56	
Median (IQR)	112.9 (63.7-174.1)	151.1 (93.6-219.7)	**.013**
L1 SAT, cm^2^			
No.	159	42	
Median (IQR)	98.1 (64.4-146.0)	104.5 (71.1-130.4)	.983

Data are presented as median (interquartile range), unless otherwise indicated. Boldface indicates a *P* value with statistical significance. IQR = interquartile range; SAT = subcutaneous adipose tissue; VAT = visceral adipose tissue.

Univariate Cox proportional hazards regression analyses demonstrated that pectoralis muscle CSA, L1 muscle CSA, and L1 VAT CSA were associated with 30-day in-hospital mortality (pectoralis muscle CSA: HR, 0.97 [95% CI, 0.95-0.99]; L1 muscle CSA: HR, 0.99 [95% CI, 0.97-1.00]; and L1 VAT CSA: HR, 1.00 [95% CI, 1.00-1.01]) (Table 3, model 1). In the multivariate Cox regression model adjusted for the 4C Mortality Score, pectoralis muscle CSA remained associated significantly with 30-day in-hospital mortality (4C Mortality Score: HR, 1.4 [95% CI, 1.3-1.4]; pectoralis muscle CSA: HR, 0.98 [95% CI, 0.96-1.00]) (Table 3, model 2). The sensitivity analyses (e-Tables 1-4) similarly demonstrated that pectoralis muscle CSA was associated significantly with 30-day in-hospital mortality as well as with 30-day overall mortality.

**Table 3:** CT Scan-Derived Body Composition Values as Predictors for 30-Day In-Hospital Mortality

Variable	Model 1		Model 2	
	HR (95% CI)	*P* Value	HR (95% CI)	*P* Value
Pectoralis muscle, cm^2^	0.97 (0.95-0.99)	**.002**	0.98 (0.96-1.00)	**.038**
L1 muscle, cm^2^	0.99 (0.97-1.00)	**.056**	0.99 (0.98-1.00)	.080
L1 VAT, cm^2^	1.00 (1.00-1.01)	**.003**	1.00 (1.00-1.00)	.451
L1 SAT, cm^2^	1.00 (0.99-1.00)	.643	…	…

Univariate Cox proportional hazards regression analysis (model 1) and multivariate analysis adjusted for 4C Mortality Score (model 2). Boldface indicates a *P* value with statistical significance. HR = hazard ratio; L1 = first lumbar vertebra; SAT = subcutaneous adipose tissue; VAT = visceral adipose tissue.

Explorative analyses allowed for an adjusted 4C Mortality Score to be constructed using age- and sex-specific quartiles for pectoralis muscle CSA. After inspecting the HRs (e-Table 5), patients with pectoralis muscle CSA less than the 25th percentile were appointed two additional points. This adjusted 4C Mortality Score (range, 0-24 points) had an AUC of 0.808 (95% CI, 0.766-0.851), which was not significantly different from the initial 4C Mortality Score (AUC, 0.806; 95% CI, 0.765-0.848; *P* = .750).

## Discussion

This large retrospective, multicenter cohort analysis demonstrated that CT scan-derived low pectoralis muscle CSA, high VAT CSA, and low muscle CSA at the L1 level were associated with higher in-hospital 30-day mortality in patients with COVID-19. Additionally, in a multivariate analysis, pectoralis muscle CSA was associated significantly with in-hospital 30-day mortality, independent of the 4C Mortality Score.

These muscle-related findings are in line with existing literature, where multiple studies have demonstrated that low CT scan-derived muscle mass, assessed at different anatomic levels, significantly predict mortality and worse clinical outcome in patients with COVID-19.^14^^,^^22^, ^23^, ^24^, ^25^, ^26^ Our data also demonstrated a significant association of high VAT with 30-day in-hospital mortality; however, with an HR of 1.00, the clinical relevance of this statistically significant association should be questioned. Additionally, when adjusted for the 4C Mortality Score, VAT was not associated significantly with mortality. This is in line with recent meta-analyses showing a significant positive association between VAT as well as obesity and COVID-19 severity, but not mortality.^15^^,^^27^

The predictive components of the 4C Mortality Score demonstrated that an older age, male sex, and having multiple comorbidities increased the risk of mortality in patients with COVID-19. The fact that low pectoralis CSA remains significantly associated with in-hospital 30-day mortality when adjusting for the 4C Mortality Score therefore is an important finding in this study. Pectoralis muscle CSA has been shown to be associated with third lumbar vertebra muscle CSA, which is linearly related to whole-body muscle mass assessed via MRI.^11^^,^^13^ Low total muscle mass is indicative for sarcopenia and is well known to be associated with mortality.^28^, ^29^, ^30^ Especially elderly people with comorbidities are more prone to sarcopenia, and as such have a higher risk for mortality.^31^ Although the exact underlying mechanisms for this association are not fully clear, it can be speculated that a higher muscle mass reflects an increased metabolic reserve, which during periods of acute catabolic disease, such as a COVID-19 infection, can protect whole-body functioning.

Whereas previously the association of CT scan-derived muscle and adipose tissue CSA with disease severity and mortality in COVID-19 was demonstrated in research settings, determining these body composition parameters in an acute clinical setting is still not applied easily in daily practice. Clinical parameters as included in the highly predictive 4C Mortality Score are already part of standard patient assessment in daily practice, and therefore are readily available. However, some of these clinical parameters inherently also are associated with CT scan-derived muscle and adipose tissue parameters.^32^ Therefore, we investigated if CT scan-derived muscle and adipose tissue CSA are still associated with mortality when adjusted for a validated set of clinical parameters, eg, the 4C Mortality Score. Based on our data, we can conclude that only pectoralis muscle CSA remains associated with 30-day in-hospital mortality when adjusted for the 4C Mortality Score. Additional sensitivity analyses (e-Tables 1-5) demonstrated no difference in this outcome when using only hospitalized patients compared with the current population, which showed an admission rate of 82.5%. Whether pectoralis muscle CSA improves the predictability of the already highly predictive 4C Mortality Score and as such needs to be added to the 4C Mortality Score was not the main question of this study. However, we constructed an adjusted 4C Mortality Score including low pectoralis CSA. In comparative AUC analyses, addition of the pectoralis muscle to the 4C Mortality Score demonstrated a small, yet statistically insignificant, improvement to an already very well-performing score. This further underlines our findings that, despite the HR of 0.98, pectoralis muscle is associated significantly with 30-day in-hospital mortality. To investigate this further, a larger dataset as well as a validation dataset of comparable magnitude as the development and validation cohort of the initial 4C Mortality Score is warranted.^17^

To add pectoralis muscle CSA to the 4C Mortality Score, cutoff values to identify patients with high or low muscle CSA are required. In line with other studies, our data demonstrated a significant association between muscle CSA and age and sex.^11^^,^^32^ However, age- and sex-specific cutoffs for pectoralis muscle CSA are still lacking. Therefore, future studies in large cohorts should focus on developing age- and sex-specific cutoff values for muscle CSA.

The current analyses were performed retrospectively on chest CT scans that were obtained with the sole purpose of assessing intrapulmonary abnormalities in an acute clinical setting. This is an important limitation that needs to be addressed, because it might have caused bias resulting from missing data, specifically regarding the extrapulmonary tissue at the L1 level (missing in 52.3% of scans). A focus on ensuring that chest CT scans include the L1 level in the future will allow for more precise (retrospective) comparison of the prognostic value of pectoralis and L1 muscle CSA.

Furthermore, the current method of semi-automated analysis of CT scan-derived muscle and adipose tissue CSA allows for retrospective analysis of CT scans at admission. This provides opportunities for long-term, longitudinal follow-up of patients with COVID-19 using the CT scans that are part of regular care. Relevant changes in muscle mass (and quality) during admission and 3 months after recovery from COVID-19 already have been described in recent studies.^33^^,^^34^ However, the current method requires trained analysts and is very labor intensive. This makes its application in the dynamic, fast-paced daily clinical practice and prognostic studies nearly impossible. Therefore, fully automated segmentation and analysis of CT scan-derived body composition through artificial intelligence algorithms has sparked attention. Both Goehler et al^35^ and Hosch et al^36^ demonstrated the potential of its use in COVID-19 by using an artificial intelligence algorithm to investigate the association of different CT scan-derived muscle and adipose tissue parameters on disease severity and mortality. Additionally, in acute trauma settings, the use of a deep learning algorithm to assess CT scan-derived muscle and adipose tissue recently was validated.^37^^,^^38^ Complete automation of this process provides the potential to move these analyses from research settings to daily clinical practice in different areas of both acute and chronic care.^39^

## Interpretation

Low CT scan-derived pectoralis muscle, high VAT, and low muscle CSA at L1 are statistically significantly associated with higher 30-day in-hospital mortality in patients with COVID-19. Additionally, CT scan-derived pectoralis muscle CSA remains associated with 30-day in-hospital mortality in patients with COVID-19 independent of the clinical 4C Mortality Score.

## Funding/Support

A research fellowship awarded to R. J. H. C. G. B. by the European Society of Clinical Nutrition and Metabolism and a grant from ZonMw [Project no. 100430040211004] awarded to H. A. G. and A. M. W. J. S. funded this work.

## Financial/Nonfinancial Disclosures

The authors have reported to *CHEST* the following: J. P. v. d. B. received funding from Amgen that was not related to this work. None declared (S. I. J. v. B., H. A. G., P. M. S., H. R. G., D. G., F. H. M. v. O., A. M. W. J. S., R. J. H. C. G. B.).

## Supplementary Data

e-Online Data
