# Supplementary material for: CT Scan-Derived Muscle, But Not Fat, Area Independently Predicts Mortality in COVID-19
Source: Chest. 2023 Mar 7;164(2):314–22. doi: 10.1016/j.chest.2023.02.048 (PMC9990885; doi:10.1016/j.chest.2023.02.048)
Supplement: e-Online Data [file mmc1.docx]

**Appendix**

e-Table 1 Difference in CT-derived body composition parameters between survival and in-hospital mortality of hospitalized COVID-19 patients.

|  | **In-hospital survival  (>30 days)** | **In-hospital death (<30 days)** | **p-value** |
| --- | --- | --- | --- |
| **Pectoralis muscle, cm^2^** |  |  |  |
| N | 364 | 104 |  |
| Median (IQR) | 35.5 (28.1-43.8) | 32.6 (24.3-38.8) | **0.002** |
| **L1 Muscle, cm^2^** |  |  |  |
| N | 179 | 51 |  |
| Median (IQR) | 90.7 (74.4-111.2) | 85.7 (66.4-103.2) | **0.025** |
| **L1 VAT, cm^2^** |  |  |  |
| N | 192 | 56 |  |
| Median (IQR) | 128.3 (72.9-185.6) | 151.1 (93.6-219.7) | 0.157 |
| **L1 SAT, cm^2^** |  |  |  |
| N | 126 | 42 |  |
| Median (IQR) | 107.1 (73.2-154.6) | 104.5 (71.1-130.4) | 0.484 |

e-Table 2 CT-derived body composition values as predictors for 30-day in-hospital mortality in hospitalized COVID-19 patients.

| **Variable** | **Model 1** | | **Model 2** | |
| --- | --- | --- | --- | --- |
|  | **HR (95% CI)** | **p-value** | **HR (95% CI)** | **p-value** |
| Pectoralis muscle, cm^2^ | 0.97  (0.95-0.99) | **0.002** | 0.98 (0.96-1.00) | **0.047** |
| L1 Muscle, cm^2^ | 0.99  (0.97-1.00) | **0.015** | 0.99  (0.98-1.00) | 0.107 |
| L1 VAT, cm^2^ | 1.00  (1.00-1.01) | **0.056** | 1.00 (1.00-1.00) | 0.600 |
| L1 SAT, cm^2^ | 1.00  (0.99-1.00) | 0.269 |  |  |

e-Table 3 Difference in CT-derived body composition parameters between survival and overall mortality of all COVID-19 patients.

|  | **Survival (>30 days)** | **Death  (<30 days)** | **p-value** |
| --- | --- | --- | --- |
| **Pectoralis muscle, cm^2^** |  |  |  |
| N | 454 | 117 |  |
| Median (IQR) | 35.5 (27.2-44.4) | 32.6 (24.7-38.8) | **0.002** |
| **L1 Muscle, cm^2^** |  |  |  |
| N | 225 | 58 |  |
| Median (IQR) | 88.3 (72.1-108.6) | 84.4 (66.1-103.7) | 0.052 |
| **L1 VAT, cm^2^** |  |  |  |
| N | 242 | 65 |  |
| Median (IQR) | 112.9 (63.9-173.8) | 146.6 (72.2-220.1) | **0.042** |
| **L1 SAT, cm^2^** |  |  |  |
| N | 156 | 45 |  |
| Median (IQR) | 101.8 (64.8-146.9) | 100.7 (70.6-127.6) | 0.684 |

e-Table 4 CT-derived body composition values as predictors for 30-day overall mortality in COVID-19 patients.

| **Variable** | **Model 1** | | **Model 2** | |
| --- | --- | --- | --- | --- |
|  | **HR (95% CI)** | **p-value** | **HR (95% CI)** | **p-value** |
| Pectoralis muscle, cm^2^ | 0.97  (0.96-0.99) | **0.002** | 0.98 (0.96-1.00) | **0.041** |
| L1 Muscle, cm^2^ | 0.99  (0.98-1.00) | **0.029** | 0.99  (0.98-1.00) | **0.039** |
| L1 VAT, cm^2^ | 1.00  (1.00-1.01) | **0.008** | 1.00 (1.00-1.00) | 0.727 |
| L1 SAT, cm^2^ | 1.00  (0.99-1.00) | 0.435 |  |  |

Note: After using a forward selection likelihood ratio approach including both pectoralis muscle and L1 muscle, the best model included pectoralis muscle adjusted for 4C Mortality Score and excluded L1 muscle.

*Table 5. Adjusted 4C Mortality Score based on adding age- and gender-specific Pectoralis Muscle CSA quartiles to the initial 4C Mortality Score.*

|  | **Model 1 (4C Mortality Score)** |  | **Model 2 (4C Mortality Score + Pectoralis)** |  |
| --- | --- | --- | --- | --- |
|  | HR (95% CI) | p-value | HR (95% CI) | p-value |
| **Age** |  |  |  |  |
| <50 years | -* |  | -* |  |
| 50-60 years | REF | - | REF | - |
| 60-70 years | 0.75 (0.23-2.39) | 0.625 | 0.76 (0.24-2.42) | 0.635 |
| 70-80 years | 3.78 (1.44-9.94) | **0.007** | 3.99 (1.51-10.55) | **0.005** |
| >80years | 8.25 (3.06-22.26) | **<0.001** | 8.27 (3.05-22.43) | **<0.001** |
| **Sex** |  |  |  |  |
| Female | REF | - | REF | - |
| Male | 1.46 (0.93-2.29) | 0.102 | 1.48 (0.93-2.35) | **0.096** |
| **Comorbidities** |  |  |  |  |
| 0 comorbidities | REF | - | REF | - |
| 1 comorbidity | 1.50 (0.65-3.44) | 0.339 | 1.45 (0.63-3.36) | 0.388 |
| > 2 comorbidities | 2.00 (0.95-4.21) | 0.068 | 1.84 (0.87-3.89) | 0.112 |
| **Respiratory Rate** |  |  |  |  |
| <20 breaths/min | REF | - | REF | - |
| 20-30 breaths/min | 1.36 (0.86-2.17) | 0.192 | 1.42 (0.88-2.28) | 0.148 |
| >30 breaths/min | 2.06 (1.16-3.63) | **0.013** | 2.06 (1.14-3.71) | **0.016** |
| **Saturation** |  |  |  |  |
| >92% | REF | - | REF | - |
| <92% | 1.91 (1.28-2.85) | **0.002** | 1.81 (1.20-2.73) | **0.004** |
| **GCS** |  |  |  |  |
| 15 | REF | - | REF |  |
| <15 | 1.24 (0.76-2.02) | 0.396 | 1.22 (0.73-2.05) | 0.442 |
| **Lab urea** |  |  |  |  |
| urea <7 | REF | - | REF | - |
| urea 7-14 | 1.36 (0.85-2.17) | 0.204 | 1.27 (0.79-2.06) | 0.329 |
| urea >14 | 1.62 (0.91-2.90) | 0.102 | 1.52 (0.83-2.77) | 0.175 |
| **Lab CRP** |  |  |  |  |
| CRP <50 | REF | - | REF |  |
| CRP 50-100 | 1.01 (0.59-1.75) | 0.961 | 1.02 (0.59-1.77) | 0.931 |
| CRP >100 | 1.47 (0.89-2.43) | 0.133 | 1.41 (0.85-2.34) | 0.185 |
| **Pectoralis quartiles** |  |  |  |  |
| >75^th^ perc. | - | - | REF | - |
| 50-75 perc. | - | - | 1.11 (0.60-2.06) | 0.749 |
| 25-50 perc. | - | - | 1.18 (0.65-2.13) | 0.581 |
| <25th perc. | - | - | 1.72 (0.98-3.01) | 0.058 |
